# Synthesis and Characterization of Calcium Alginate-Based Microspheres Entrapped with TiO_2_ Nanoparticles and Cinnamon Essential Oil Targeting Clinical *Staphylococcus aureus*

**DOI:** 10.3390/pharmaceutics14122764

**Published:** 2022-12-09

**Authors:** Tayyaba Zaineb, Bushra Uzair, Waleed Y. Rizg, Waleed S. Alharbi, Hala M. Alkhalidi, Khaled M. Hosny, Barkat Ali Khan, Asma Bano, Mohammed Alissa, Nazia Jamil

**Affiliations:** 1 Department of Biological Sciences, International Islamic University, Islamabad 44000, Pakistan; 2 Department of Pharmaceutics, Faculty of Pharmacy, King Abdulaziz University, Jeddah 21589, Saudi Arabia; 3 Center of Innovation in Personalized Medicine (CIPM), 3D Bioprinting Unit, King Abdulaziz University, Jeddah 21589, Saudi Arabia; 4 Department of Clinical Pharmacy, Faculty of Pharmacy, King Abdulaziz University, Jeddah 21589, Saudi Arabia; 5 Drug Delivery and Cosmetics Lab (DDCL), GCPS, Faculty of Pharmacy, Gomal University, Dera Ismail Khan 29050, Pakistan; 6 Department of Microbiology, University of Haripur, Haripur 22620, Pakistan; 7 Department of Medical Laboratory Sciences, College of Applied Medical Sciences, Prince Sattam Bin Abdulaziz University, Al-Kharj 11942, Saudi Arabia; 8 Department of Microbiology & Molecular Genetics, Punjab University, Lahore 54000, Pakistan

**Keywords:** microspheres, *Staphylococcus aureus*, essential oils, metal oxides, *Nigella sativa*, cinnamon essential oil, antibiotic activity, flow cytometry, biofilm inhibition analysis

## Abstract

It is important to create new generations of materials that can destroy multidrug-resistant bacterial strains, which are a serious public health concern. This study focused on the biosynthesis of an essential oil entrapped in titanium dioxide (TiO_2_) calcium alginate-based microspheres. In this research, calcium alginate-based microspheres with entrapped TiO_2_ nanoparticles and cinnamon essential oil (CI-TiO_2_-MSs) were synthesized, using an aqueous extract of *Nigella sativa* seeds for TiO_2_ nanoparticle preparation, and the ionotropic gelation method for microsphere preparation. The microspheres obtained were spherical, uniformly sized, microporous, and rough surfaced, and they were fully loaded with cinnamon essential oil and TiO_2_ nanoparticles. The synthesized microspheres were analyzed for antibacterial activity against the clinical multidrug-resistant strain of *Staphylococcus aureus*. Disc diffusion and flow cytometry analysis revealed strong antibacterial activity by CI-TiO_2_-MSs. The synthesized CI-TiO_2_-MSs were characterized by the SEM/EDX, X-ray diffraction, and FTIR techniques. Results showed that the TiO_2_ nanoparticles were spherical and 99 to 150 nm in size, whereas the CI-TiO_2_-MSs were spherical and rough surfaced. Apoptosis analysis and SEM micrography revealed that the CI-TiO_2_-MSs had strong bactericidal activity against *S. aureus*. The in vitro antibacterial experiments proved that the encapsulated CI-TiO_2_-MSs had strong potential for use as a prolonged controlled release system against multidrug-resistant clinical *S. aureus*.

## 1. Introduction

In recent years, intensifying bacterial infections, particularly antibiotic-resistant bacterial infections, have become a threat to the general well-being of people around the world. Antibiotic use began in the “golden era” from 1940 to 1960, following the discovery of penicillin by Alexander Fleming. During this era, more widely used antibiotics were discovered [1,2]. Antibiotic-resistant bacteria were already well known more than 50 years ago since, by the late 1950s, the majority of penicillin, which was once typically used to treat *S. aureus*, became ineffective against them [3]. In the previous century, bacterial resistance to antibiotics was found to be related to an intrinsic resistance of bacteria to antimicrobial pathogens and also to increased resistance from other bacterial strains through the processes of gene mutation and gene transfer [4,5]. Many bacterial strains that were resistant to multiple drugs (i.e., multidrug-resistant (MDR) strains or superbugs) developed through mutations combined with years of antibiotic selection, causing them to be untreatable [6]. Efforts were then made to look for new antibiotics that could attack these superbugs, but the efforts were slowed by the rapid formation of new antibiotic-resistant strains. MDR strains can have harmful consequences because traditional antibiotics are increasingly losing their potency in the fight against these bacteria [7]. To increase the efficacy of current antibiotics, nanoparticles have been increasingly used discreetly as efficient antimicrobial agents. They have been used in the form of metal oxides and metal nanoparticles or combined with delivery platforms such as solid lipid nanoparticles, polymersomes, and liposomes. Although most current antibiotic agents target bacteria by a common mechanism, nanoparticles of a metal or a metal oxide, which have an inherent antiseptic effect, can attack pathogenic bacteria by using different mechanisms at the same time [8]. Nanoparticles of a metal and a metal oxide can impact cell walls and intracellular cells simultaneously through direct interactions, the release of ions, and the production of reactive oxygen species (ROS) [9]. Among the most serious threats to the successful management of microbial infections is the superbug. Over the years, volatile oils and certain other extracts of plants have elicited interest as sources of organic products. These have been studied as natural treatments for the prevention of multiple bacterial infections [10]. The World Health Organization has recognized that a substantial portion of the world’s population depends on traditional drugs for primary health care. Aromatic and herbal plant species are widely used for medicinal purposes and are a major source of natural organic molecules.

One of the most notable new areas of research in this century is nanotechnology. It deals with the planning, development, investigation, and use of systems on a nanoscale. Biotechnology, which provides a way of understanding biological systems and using expertise for industrial development, is another fascinating scientific discipline of today. These two research areas meet in nanobiotechnology. Nanotechnology and biotechnology are used to analyze and design nanobiosystems to address a broad range of challenges and develop a wide range of applications [11].

Titanium dioxide (TiO_2_), a versatile transition metal oxide, is naturally present as an oxide of the metal titanium. Anatase, brookite, and rutile forms of TiO_2_ occur in nature. The refractive index of these phases is high (anatase = 2.488, brookite = 2.583, rutile = 2.609), it has high chemical and thermal stability, and has reduced absorption and reduced dispersion in visible and near-infrared spectral ranges [12]. TiO_2_ nanoparticles are manufactured in huge amounts worldwide for use in a wide variety of applications [13]. Much research has concentrated on the use of titanium in technology and medicine, including as an antibacterial agent, as well as for dye-sensitized solar cells, photodynamic applications, and wastewater treatments [14].

Essential, or volatile, oils are sweet-smelling oily liquids extracted from different parts of plants and are labeled Generally Recognized as Safe by the United States Food and Drug Administration [15]. It has been shown that essential oils have antioxidant, insecticidal, antiviral, antifungal, and antibacterial properties [15,16].

Microspheres are spherical (ranging from 1 to 1000 μm in size) microscopic structures made from a support substance consisting of a matrix in which the compound of the drug is dispersed. The materials comprising the matrix are most often natural or synthetic polymers. They must be nontoxic in the conditions for which they are to be used and absorbable if they are intended for systemic application [17,18]. In recent decades, there has been significant interest in the encapsulation of essential oils and the development of biodegradable microparticles (i.e., microcapsules and microspheres). Their encapsulation helps to conserve and protect their usable properties and to modulate their release. 

Among the various biopolymers that use the processing of microparticles, the sodium salt of alginic acid is of great value as it is biocompatible, biodegradable, and nontoxic. Alginic acids are naturally occurring, harmless, ecofriendly polysaccharides obtained from brown seaweed. They are straight polymers comprising the residues of 1,4-linked-*D*-mannuronate and α-l-guluronate, which solidify in the presence of divalent cations such as Ca^2+^ ions, owing to the stockpiling of residues of guluronate (G), as well as the formation of a calcium-connected “egg box linkage” [19]. 

As alginates are ecofriendly, biocompatible, and muco-adhesive, they have a special significance in many biological and medical applications, particularly as drug carriers [19]. Alginic acids are, however, hemocompatible and have not been shown to persist in any large organ, although they do exhibit signs of depletion in vivo [20]. Sodium alginate is used in several oral and topical medicinal preparations. Unlike other more traditional solvent-based systems, it has been primarily used for aqueous drug microencapsulation [20,21].

By considering all the above factors, surface-modified TiO_2_ nanoparticles with cinnamon essential oil (CI) entrapped in sodium alginate microspheres (MSs) were developed. The characteristics of the synthesized microspheres, such as their size, morphology, crystallinity, and chemical structure, were determined, and the antibacterial and antibiofilm activity against the clinical MDR *S. aureus* strain was confirmed using various standard antibacterial assays, including membrane damage analysis by a flow cytometer. 

## 2. Materials and Methods

### 2.1. Bacterial Strains

A clinical bacterial strain from a patient suffering from bacteremia caused by *S. aureus* was obtained from the Clinical Laboratory of Microbiology at the Pakistan Institute of Medical Sciences (PIMS), Islamabad, Pakistan. The strain was preserved in glycerol. An amount of 0.5 mL of the log-phase cells of the clinical *S. aureus* was placed in Luria broth and 0.5 mL of glycerol in a sterile Eppendorf tube and stored at minus 70 °C.

### 2.2. Antibiotic Susceptibility Testing

The antibiotic susceptibility was determined using the disc diffusion assay. *S. aureus* was placed on a sterile Petri plate containing Mueller–Hinton (BIO-RAD) agar medium. Six antibiotic paper discs were prepared with a fixed amount of ampicillin (10 μg), ofloxacin (5 μg), ciprofloxacin (5 μg), moxifloxacin (5 μg), or levofloxacin (5 μg). The Petri plate was incubated overnight at 37 °C before the results were determined. Zone diameters were calibrated for each disc according to the guidelines issued by the Clinical and Laboratory Standards Institute (CLSI) [22,23].

### 2.3. Screening of Essential Oils for Synthesis of Modified Microspheres

For the screening of essential oils, an agar well diffusion technique was used. Twelve essential oils were purchased from the D. Watson Blue Area drug store in Islamabad, Pakistan. They were oils of rose, cedar wood, neem, clove, cinnamon, lemon, garlic, ginger, eucalyptus, omum seed, jojoba, and black cumin seed. In an agar well diffusion procedure to obtain a semiconfluent growth of clinical *S. aureus*, 50 µL of an overnight culture of clinical *S. aureus* was swabbed on a sterile Mueller–Hinton agar plate, and a well of 6 mm in width was pierced using a sterile metal borer. A total of 50 μL of each essential oil was placed in each well, and the inoculated plates were incubated at 37 °C [24].

### 2.4. Biosynthesis of TiO_2_ Nanoparticles Using Extract of Nigella sativa Seed

#### 2.4.1. Preparation of Extract of *Nigella sativa* Seed

*Nigella sativa* seeds were obtained from the Mohammadi Dawakhana and Pansar Store G-9, Markaz, Islamabad. Seed authentication was carried out by Dr. Ibrar Shinwari, a taxonomist from the Department of Environmental Sciences at International Islamic University. In the first stage of synthesis, the dried seeds were ground to a very fine powder, and 50 g of this powder was added to 500 mL of sterile double-distilled water. The solution was placed in an incubator at 50 °C and stirred at 160 rpm for 24 h. The obtained extract was filtered first with a Gaussian filter and then with Whatman filter paper grade 1. The filtered extract was stockpiled at 4 °C for further use.

#### 2.4.2. GCMS of *Nigella sativa* Seed Extract

GCMS analysis was carried out [25] in order to determine whether phytochemical compounds were present in the aqueous extract of *N. sativa*. The fine powder, 10 g, was dissolved in 30 mL of ethanol for 24 h and filtered using a 4 µm syringe filter, and 2 µL of the concentrate was used for GCMS evaluation. An Agilent device (Agilent Technologies Japan Ltd., Tokyo, Japan) was used in the analysis, and the furnace was kept at 110 °C, for 2 min. The injector temperature was set at 250 °C, and the spectral data were obtained at 70 EV; the scan interval was 0.5 s, and the identification of the microspheres was carried out for 30 min. The average peak area was compared with the total area of each phytochemical component to determine its relative abundance. Dr. Duke’s Phytochemical and Ethnobotanical Databases of Dr. James Duke of the Agricultural Research Service at the United States Department of Agriculture were used to identify the phytochemical components. The unknown and known spectrums of the components were compared using the National Institute of Standards and Technology database to interpret the GCMS data. The name, molecular structure, and molecular weight of each component were determined [26].

#### 2.4.3. Biosynthesis of TiO_2_ Nanoparticles

The TiO_2_ nanoparticles were prepared as described earlier [27]. A total of 10 mL of powdered seed extract was mixed with 90 mL of 5 mM aqueous solution of orthotitanate (Oxoid Ltd., Basingstoke, Hampshire, UK). The mixture was stirred for 2 h by a magnetic stirrer (SCILOGEX LLC, Rocky Hill, CT, USA) at 65 °C at 150 rpm. To purify the TiO_2_ nanoparticles, centrifugation was carried out at 3000 rpm for 15 minutes using a GC Bio Tech centrifuge (GC Bio Tech, Cambridge, UK), and the solution was placed in a dry oven (DHG 9053a) at 60 °C for 1 h. The nanoparticles were calcined at 500 °C for 3 h in a furnace and stored at 4 °C for further use.

### 2.5. Synthesis of Calcium Alginate-Based Microspheres

**Figure 1:**
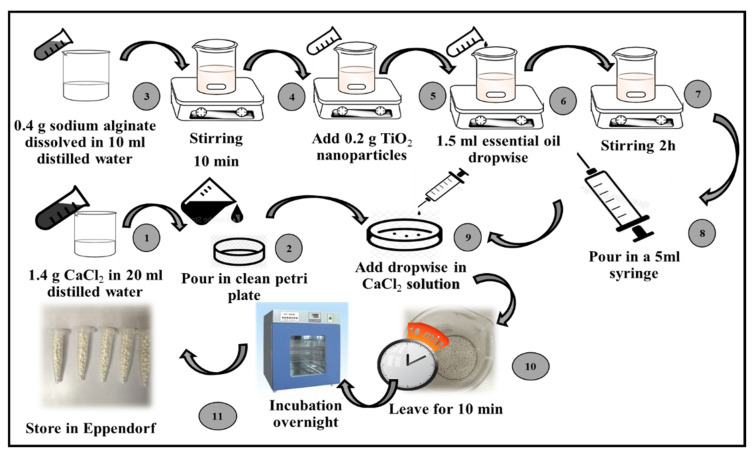
Biosynthesis of microspheres containing surface-modified essential oil-conjugated TiO_2_ nanoparticles.

Microspheres were prepared using three main steps [28] (Figure 1).

(a) Preparation of CaCl_2_ solution: A CaCl_2_ solution was prepared by dissolving 1.4 g of CaCl_2_ (Oxoid Ltd.) in 20 mL of sterile distilled water. The obtained solution was kept for 10 min at room temperature.
(b) Incorporation of TiO_2_ nanoparticles and essential oil in calcium alginate: A total of 0.4 g of sodium alginate (Oxoid Ltd.) was dissolved in 10 mL of double-distilled water and stirred for about 10 min. Then, 0.2 g of the TiO_2_ nanoparticles and 1.5 mL of the selected essential oil (i.e., the cinnamon essential oil) were added separately in a dropwise manner.
(c) Formation of microspheres by ionotropic gelation method: The prepared TiO_2_ nanoparticles and cinnamon essential oil with calcium alginate incorporated was drawn up in a 5 mL syringe with a 22-gauge needle and added dropwise to a CaCl_2_ solution. The synthesized microspheres were kept in a CaCl_2_ solution for about 10 min.
(d) Drying of microspheres: The microspheres were rinsed with distilled water and desiccated in a drying oven for 24 h at 45 °C. The synthesized microspheres were stored at 4 °C in microcentrifuge tubes for further use.

### 2.6. Characterization of Purified TiO_2_ Nanoparticles and Modified Microspheres

The surface morphologies of the purified TiO_2_ nanoparticles and the essential oil-conjugated TiO_2_ nanoparticle-containing microspheres (i.e., the CI-TiO_2_-MSs) were examined using scanning electron microscopy (SEM Hitachi S–2400, Tokyo, Japan). An acceleration voltage of 5 kV and magnification range of 25 to 10 k were used. The microspheres were mounted on the aluminum stub with the help of adhesive double-sided carbon tape, sputter coated with 20 nm gold film, and examined in the scanning electron microscope. A few beads were cut with a sterile medical blade for cross-sectional examinations [29,30]. Broad-angle X-ray diffractograms of the purified TiO_2_ nanoparticles and essential oil-conjugated TiO_2_ nanoparticles were calculated using a D8 Advance Bruker with Bragg–Brentano geometry with a copper sealed tube ray source producing Cu kα irradiation at a wavelength of 1.54 Å from a generator operating at 60 kV and 60 mA. For data collection, detector scans were used at grazing incidence angles ranging from 10° to 80°. The samples were then analyzed [31]. FTIR spectrums of the purified TiO_2_ nanoparticles and modified microspheres were recorded by means of the Thermo/Nicolet MAGNA-IR 560 (Champaign, IL, USA), at 500 to 4000 cm^−1^ to determine qualitatively the IR-active functional groups of TiO_2_ nanoparticles and modified microspheres. For the FTIR sample preparation, 0.02 g of the TiO_2_ nanoparticles was ground with 0.2 g of potassium bromide and pressed into a pellet form using a desktop powder presser/dry pressing machine (EQ-YLJ-24T (MTI, Seoul, Korea)) [31].

### 2.7. Antibacterial Activity of Modified Microspheres

The antibacterial activity of the modified CI-TiO_2_-MSs was screened by the standard disc diffusion method [32]. The synthesized microspheres were placed on MH agar plates inoculated with an overnight culture of the clinical strain of *S. aureus*, and the inoculated plates were incubated at 37 °C for 24 h. At the end of the incubation period, the zones of inhibition were measured in millimeters.

### 2.8. Detection of Membrane-Damaging Potential by Flow Cytometry

The membrane-damaging potential of the CI-TiO_2_-MSs was measured by flow cytometric analysis using the Annexin-V-FITC and propidium iodide stains (the Annexin-V binding buffer, propidium iodide, and Annexin-V-FITC kit were obtained from Biolegend, San Diego, CA, USA). Approximately 3 × 10^5^ cells of clinical *S. aureus* were treated with a synthesized 10 mg of CI-TiO_2_-MSs at 37 °C for 10 h. After the treatment period, the cells were centrifuged and washed with phosphate buffer (PBS, Sigma-Aldrich, St. Louis, MO, USA). The treated and untreated cells of *S. aureus* were stained with 2.5 µL of propidium iodide and 2.5 µL of Annexin-V and kept for 30 min at 25 °C in the dark. The intensities of the Annexin-V-FITC and propidium iodide staining were recorded using a FACScan flow cytometer (Beckman Coulter Cytomics FC500, Chaska, MN, USA) [33].

### 2.9. Observation of Morphological Changes in Staphylococcus aureus Cells

Morphological changes in *Staphylococcus aureus* were analyzed by scanning electron microscopy (SEM) as previously described [34]. Treated and untreated (control) bacterial cells were fixed in 2.5% glutaraldehyde in 0.1 M PBS (at pH 7.0) for 2 h at 4 °C, washed thrice with PBS, and then post-fixed in 1% osmium tetroxide for 1.5 h at 4 °C. The cells were then dehydrated with an ordered series of ethanol and isoamylacetate. The samples were dried with liquid CO_2_, and then mounted on SEM stubs, consequently sputter-coated with gold–palladium, and examined with a scanning electron microscope (HITACH, Model S-3400N) at an accelerating voltage of 10 kV.

### 2.10. Biofilm Inhibition Analysis

The biofilm inhibition activity of the modified microspheres was assessed using the crystal violet staining method [35]. In a Falcon tube containing Luria broth, the log-phase cells of an *S. aureus* culture were exposed to 10 mg of modified microspheres. The tube was then incubated for 48 h at 37 °C. The broth medium was decanted from the tube, and the tube was kept in an inverted position for drying. The dried tube was stained with crystal violet dye (0.1 % *w*/*v*) for 30 min. To remove the excess stain, the tube was washed three times with distilled water and inverted for drying. The tube was observed phenotypically for biofilm inhibition. If a visible purple-colored film lined the walls of the Falcon tube, the biofilm production was considered positive. Falcon tubes containing only Luria broth not inoculated with *S. aureus* were used as a negative control in the experiment. 

### 2.11. Hemolytic Activity Analysis

The biocompatibility of the synthesized microspheres was analyzed using human blood. Sterile blood agar plates using 5 % human blood and a blood agar base (Oxoid Ltd.) were prepared, and synthesized modified microspheres were placed at the center of each plate in different concentrations of 2 MSs (4 mg), 4 MSs (8 mg), and 6 MSs (12 mg). The 0.1 % Triton X (Merck, Pvt Ltd., Karachi, Pakistan) was used as a positive control, and DMSO served as a negative control. The inoculated plates were incubated overnight at 37 °C and observed for signs of hemolysis. An area of clearance around the microsphere was considered an indication of the lysis of red blood cells [36]. 

### 2.12. Statistics

GraphPad Prism was used for statistical analysis. Student’s *t*-test was used for comparing antibacterial effects. Results are stated as mean ± SD unless otherwise stated. Correlation was applied to the data and *p*-value < 0.05 was chosen as the level of significance. 

## 3. Results

### 3.1. Antibiotic Susceptibility Testing

**Figure 2:**
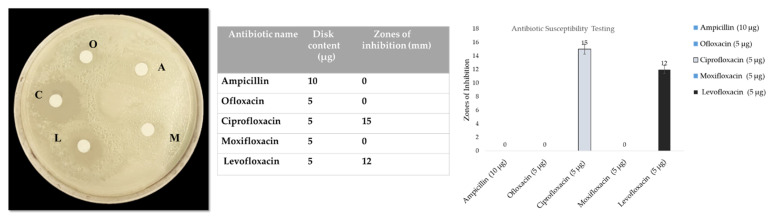
Antibiotic susceptibility testing for clinical strain of *S. aureus*.

Figure 2 shows the Kirby–Bauer disc diffusion susceptibility test for clinical *S. aureus.* The zones of inhibition were measured for five different antibiotics and compared according to the CLSI guidelines. *S. aureus* appeared to be resistant to ampicillin, oflaxacin, and moxifloxacin while ciprofloxacin and levofloxacin showed 15 mm and 12 mm zones of inhibition, respectively. When Student’s *t*-test was applied, these two antibiotics showed a statistically significant difference.

### 3.2. Screening of Essential Oils for Modified Microspheres

**Figure 3:**
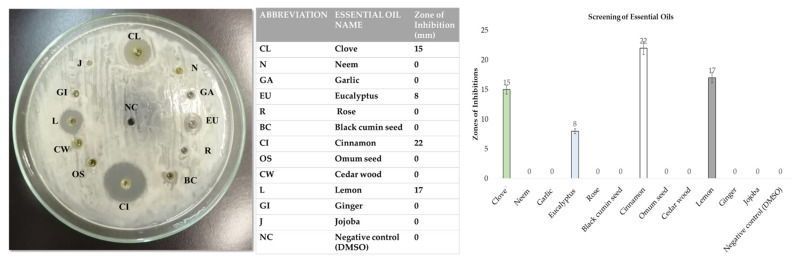
Screening of essential oils using the agar well diffusion method against *S. aureus*.

Figure 3 shows the results of the screening of essentials oils and zones of inhibition measured against *S. aureus*. The clove (15 mm), cinnamon (22 mm), eucalyptus (8 mm), and lemon (17 mm) essential oils showed zones of inhibition against *S. aureus*. The most potent activity was exhibited by the cinnamon essential oils, as seen in Figure 3, and this oil was selected for the synthesis of the essential oil-conjugated TiO_2_ nanoparticles encapsulated in calcium alginate microspheres. Statistically, the results were significantly different. 

### 3.3. GCMS of Extract of Nigella sativa Seed

**Figure 4:**
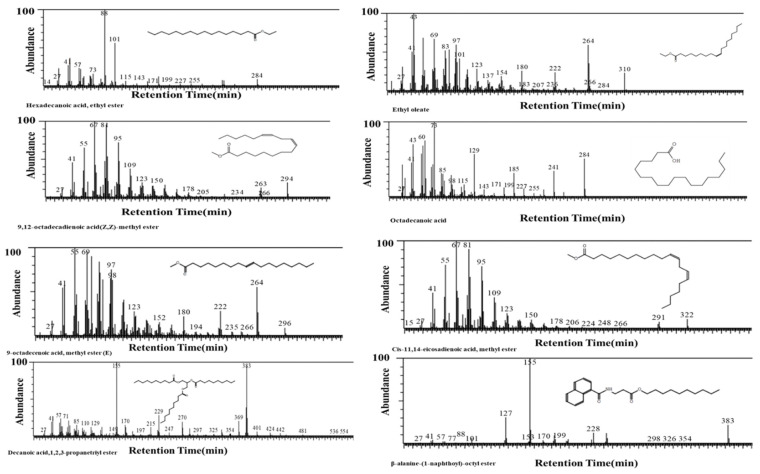
GCMS analysis of *N. sativa* seed extract.

For the identification of biochemical constituents, GCMS was carried out in an ethyl acetate extract of *N. sativa*. Eight different phytochemical components were identified, namely, hexadecanoic acid, ethyl ester (24%); 9, 12-octadecadienoic acid (*Z*,*Z*)-methyl ester (or) methyl linoleate (9%); 9-octadecenoic acid, methyl ester(E) (or) methyl eladate (5%); decanoic acid, 1,2,3-propanetriyl ester (24%); ethyl oleate (25%); octadecanoic acid (2%); cis-11,14-eicosadienoic acid, methyl ester (5%); and β-alanine-(1-naphthoyl)-octyl ester (6%). By using Duke’s Phytochemical and Ethnobotanical Databases, the activities of the phytochemical constituents were predicted. It was observed that the hexadecanoic acid, ethyl ester, and β-alanine-(1-naphthoyl)-octyl ester had antioxidant properties and could thus serve as reducing agents for the synthesis of TiO_2_ nanoparticles. In Figure 4, the percentages of the phytochemical compounds present in the extract of the *N. sativa* seed are given [25].

### 3.4. Characterization of Purified TiO_2_ Nanoparticles and Modified Microspheres

**Figure 5:**
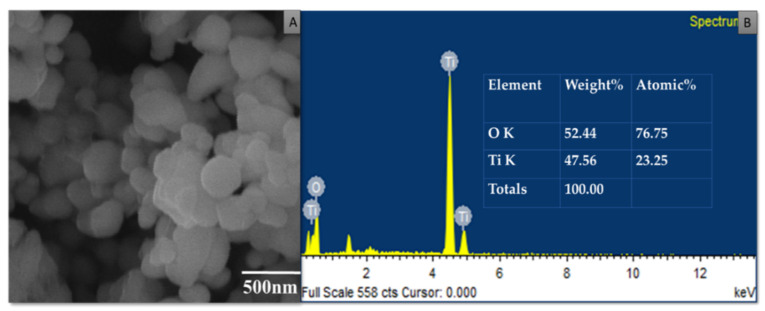
SEM/EDX of TiO_2_ nanoparticles. (**A**) SEM image of TiO_2_ nanoparticles at 500 nm. (**B**) EDX of TiO_2_ nanoparticles.

**Figure 6:**
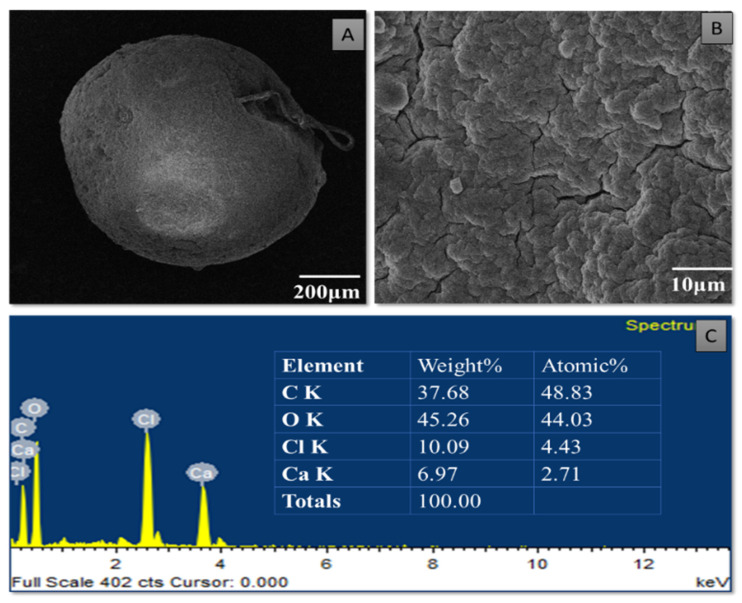
SEM/EDX of blank MS. SEM image of MS at low magnification (**A**) and at high magnification (**B**). (**C**) EDX of MS.

**Figure 7:**
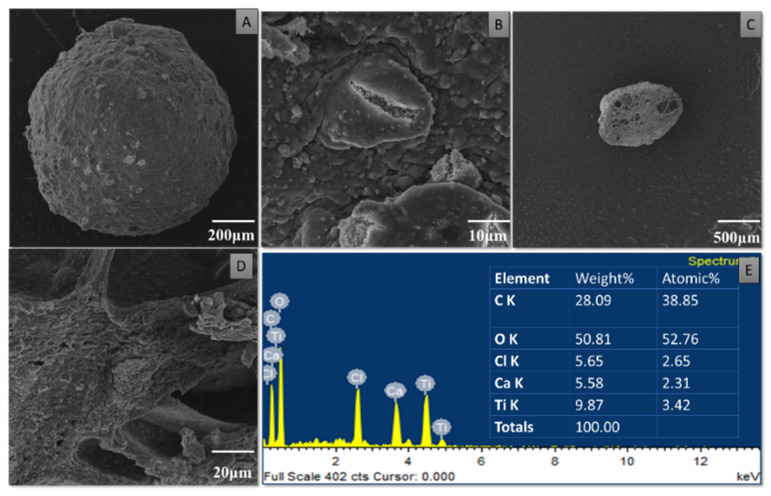
SEM/EDX of CI-TiO_2_-MS. SEM image of CI-TiO_2_-MS at low magnification (**A**) and high magnification (**B**). Cross-sectional SEM images of CI-TiO_2_-MS at low magnification (**C**) and high magnification (**D**). EDX of CI-TiO_2_-MS (**E**).

SEM micrographs of the purified TiO_2_ nanoparticles and modified microspheres are shown in Figure 5, Figure 6 and Figure 7. SEM micrographs of the synthesized TiO_2_ nanoparticles (see Figure 5) showed spherical nanoparticles that were uniform in size and evenly distributed on the surface [37]. The size of the microspheres decreased after the sintering process, but they maintained their original spherical shapes. Greater surface roughness and cracks were evident in the CI-TiO_2_-MSs (see Figure 7), but a smooth surface was observed in the unloaded microspheres (see Figure 6). A cross-sectional SEM of the CI-TiO_2_-MSs shows that the microspheres were fully loaded with the TiO_2_ nanoparticles and the essential oil (see Figure 7C,D) [38,39]. The elemental composition and contamination of samples were examined with EDX, and the results showed that the TiO_2_ nanoparticles, blank microspheres, and CI-TiO_2_-MSs were free of contamination.

**Figure 8:**
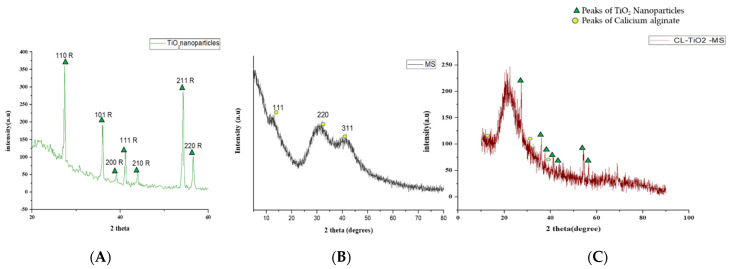
XRD peaks of (**A**) TiO_2_ nanoparticles, (**B**) blank MS, and (**C**) CI-TiO_2_-MS.

The X-ray diffraction (XRD) pattern was examined to study the phase formation and structure of the samples. According to Figure 8A, a well-crystallized rutile profile was observed for the TiO_2_ nanoparticles. Figure 8B shows the XRD patterns of the blank microspheres. Calcium alginate generally has a crystal-like structure and intermolecular hydrogen bonding between the alginate chains [40]. Three diffraction points at 111, 220, and 311 were identified for the calcium alginate. One point was due to the reflection of the 111 plane from an α-1-guluronic acid residue, and another was due to the reflection of the 220 plane from β-D-mannuronic acid [41]. Figure 8C shows the XRD pattern of the CI-TiO_2_-MSs containing rutile peaks and calcium alginate peaks.

**Figure 9:**
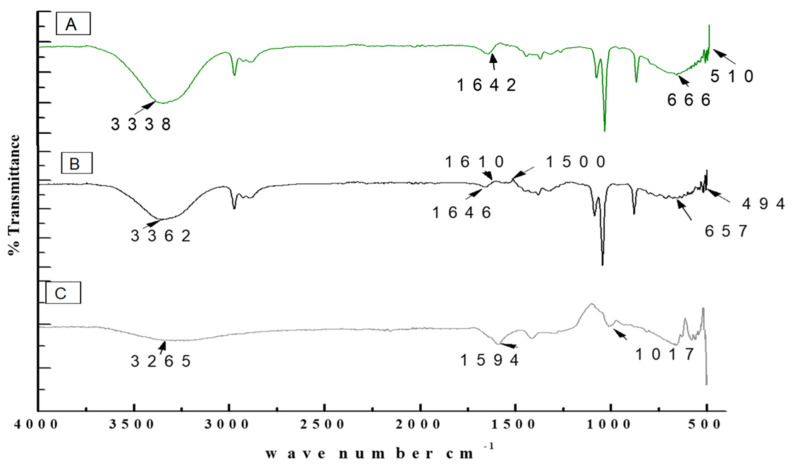
FTIR spectrums of (**A**) TiO_2_ nanoparticles, (**B**) CI-TiO_2_-MS, and (**C**) blank MS.

The FTIR spectrum was used for the chemical interpretation of the synthesized TiO_2_ nanoparticles and modified microspheres. These spectrums are shown in Figure 9. The peak values and functional group vibrational modes of the microspheres and TiO_2_ nanoparticles are given in Table 1 [42,43,44,45,46]. A bending vibration of 3416–2360 cm^−1^ is characteristic of the -OH group, and 666 and 480–521 cm^−1^ are characteristic peaks of rutile phase TiO_2_ nanoparticles. The -OH bending mode of water Ti-OH is 1642 cm^−1^. The asymmetric stretching vibration of free carboxyl groups is 1594 cm^−1^, and the stretching of the C-O-C bond in unloaded microspheres is 1017 cm^−1^. The absorption stretching of the aromatic benzene ring is 1610 cm^−1^. The stretching vibration of an aldehyde group is 1500 cm^−1^. The vibrational mode of a phenyl ring is 1442 cm^−1^, that of a –CH_3_ group is 1376 cm^−1^, and that of the –C-O-C of aromatics is 1262 cm^−1^**.**

### 3.5. Antibacterial Activity of Purified TiO_2_ Nanoparticles and Modified Microspheres

**Figure 10:**
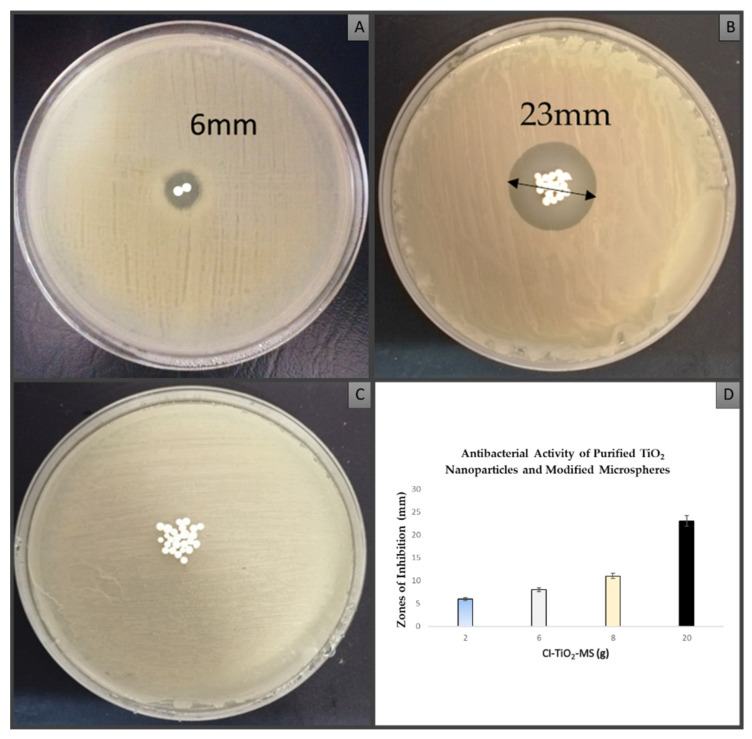
Antibacterial activity of CI-TiO_2_-MSs against *S. aureus*; (**A**) 2 MSs (**B**) and 20 MSs. (**C**) Antibacterial activity of TiO_2_-MSs. (**D**) The graph depicts increase in the zone of inhibition by increasing CI-TiO_2_-MS concentration.

Figure 10 shows the antibacterial activity in the zones of inhibition of various diameters for the CI-TiO_2_-MSs against *S. aureus* exposed to 2 or 20 microspheres.

### 3.6. Detection of Cell Membrane Damage by Flow Cytometry

**Figure 11:**
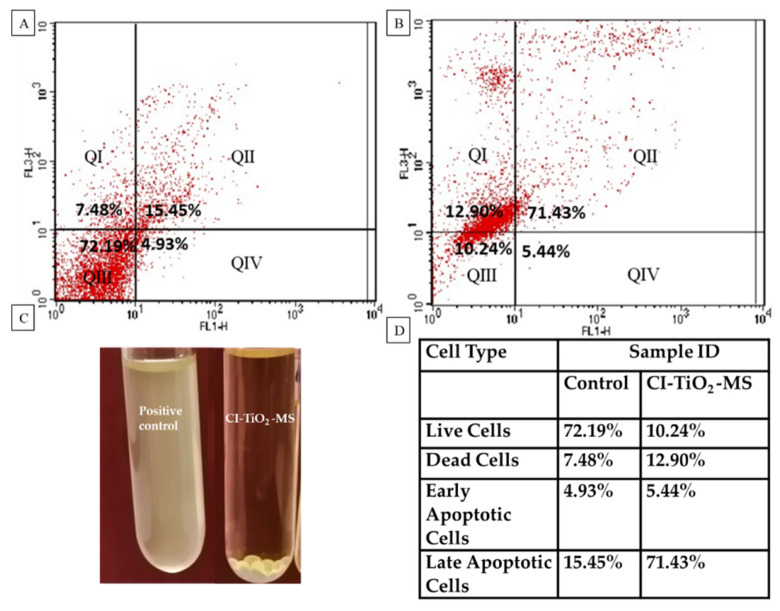
Flow cytometry results. (**A**) *S. aureus* control. (**B**) *S. aureus* treated by CI-TiO_2_–MSs. (**C**) Samples of flow cytometry. (**D**) Table showing the percentages of lysing cells.

**Figure 12:**
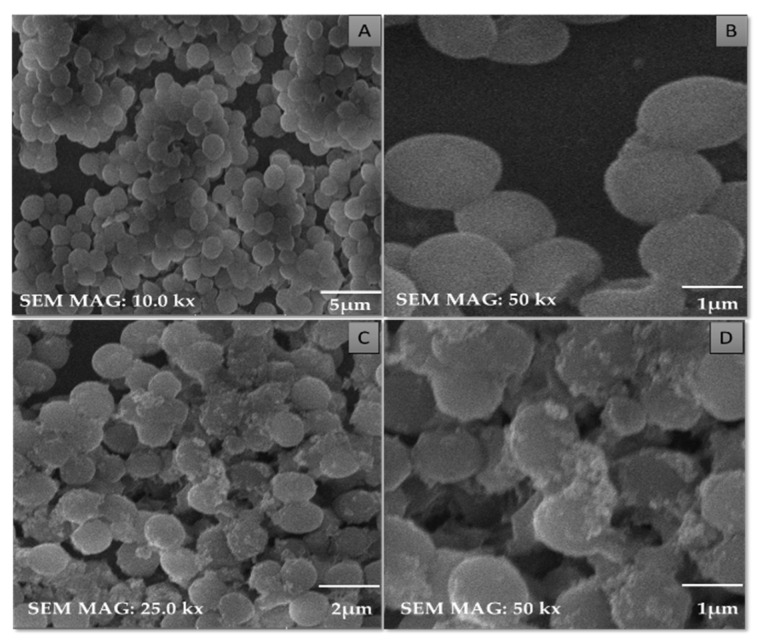
Untreated *S. aureus* at (**A**) 5 μm, (**B**) 1 μm. Action of CI-TiO_2_-MSs on *S. aureus* detected by SEM at (**C**) 2 μm, (**D**) 1 μm.

Figure 11 shows the flow cytometry data obtained after the treatment of *S. aureus* with CI-TiO_2_-MS. Figure 12B shows *S. aureus* cells treated with CI-TiO_2_-MSs having 5.44 % early apoptotic cells and 71.43 % late apoptotic cells. The results show that the cell population in the right upper quadrant, which reveals the late apoptotic cells, was larger for the CI-TiO_2_-MSs. It was confirmed that cell death caused by CI-TiO_2_-MSs showed late apoptotic features. Therefore, the cell death may have been due to membrane leakage in the clinical *S. aureus* population treated with modified microspheres.

### 3.7. Observation of Morphological Changes in Cells of Staphylococcus aureus

SEM micrographs of treated and untreated cells of *S. aureus* are shown in Figure 12. In the untreated cells, the spherical shapes of the cocci of the *S. aureus* cells were maintained (see Figure 12A–C). The structural damage is clearly seen in the cells treated with CI-TiO_2_-MSs (see Figure 12D), and the appearance is in agreement with the results of the disc diffusion and flow cytometry tests. The expected killing of *S. aureus* cells was seen after treatment with CI-TiO_2_-MSs. Furthermore, it was discovered that the shapes of the cocci were no longer maintained, and this could have been the final cause of cell death.

### 3.8. Biofilm Inhibition Analysis

**Figure 13:**
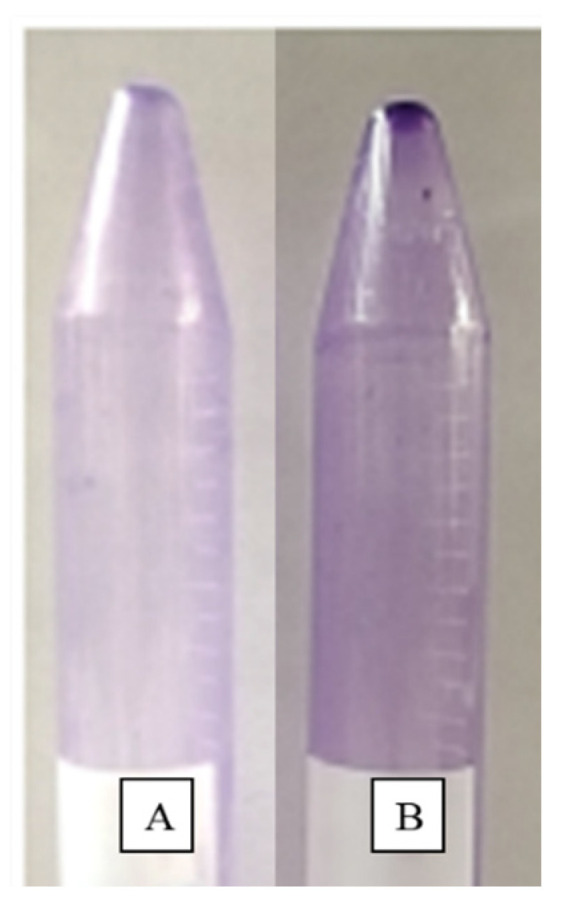
Biofilm inhibition analysis. (**A**) Positive control containing *S. aureus*. (**B**) *S. aureus* treated with CI-TiO_2_-MSs.

Figure 13 depicts the inhibition of the biofilm by crystal violet staining. Compared with the untreated cells, the treated cells showed a reduction in the intensity of the crystal violet’s color.

### 3.9. Hemolytic Activity Analysis

**Figure 14:**
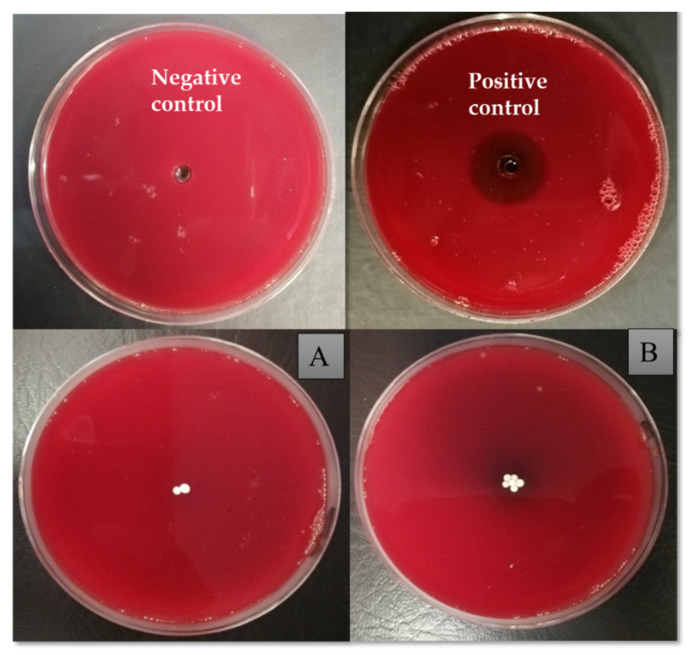
Hemolytic activity analysis on blood agar plates with DMSO (negative control) and Triton X (positive control). CI-TiO_2_-MS: (**A**) 2 g, and (**B**) 6 g.

Figure 14 shows that the CI-TiO_2_-MSs had no hemolytic action even for the quite high number of modified microspheres tested. There was no illumination or clearance of red blood cells around the microspheres on the plates exposed to two and six MSs. However, the Triton X (positive control) had a 30 mm hemolytic halo and a brightly illuminated area, and these are indications of the lysis of red blood cells. 

## 4. Discussion

In recent times, the sodium salt of alginic acid was identified as a beneficial material for pharmaceutical use, with the ability to be processed under optimal circumstances [47,48,49,50]. Easy and flexible aqueous-based sodium alginate gel formation in the presence of Ca^2+^ ions can also be used for cell immobilization [51], hemoglobin carriers [52], hybrid artificial organs [53], drug delivery systems [54], and macromolecular delivery systems [55,56,57]. The easy and flexible aqueous gel formation of the sodium salt of alginic acid in the presence of ions such as Ca^2+^ has also been used for cell immobilization [49], for hybrid artificial organs [53], hemoglobin carriers [52], macromolecular delivery systems [58,59,60], and drug-delivery systems [54]. Several trials have demonstrated that the use of nanoparticles in medications greatly increases the efficiency of current treatments. Combining antibiotics and nanoparticles increases their accumulation in infected cells, along with their ability to penetrate membranes of cells. Increasing the specificity of antibiotics is a major challenge for modern medicine. It can be achieved by working on medicinal pathways with increased specificity and reduced side effects, together with improved treatment performances [57,61]. In this scientific work, calcium alginate-based microspheres modified with cinnamon essential oil (CI-TiO_2_-MSs) were successfully synthesized by an ionotropic gelation method using a calcium alginate polymer. The biosynthesis of calcium alginate microspheres loaded with TiO_2_ nanoparticles and cinnamon essential oil was carried out for the first time in this research. The synthesized microspheres (CI-TiO_2_-MSs) were rough and spherical in shape compared with the blank microspheres. The surfaces of the unloaded microspheres were smooth, whereas the modified microspheres had rough and porous surfaces. The capacity for incorporation could be improved steadily by adding higher amounts of the sodium salt of alginic acid. The increased amount of alginate resulted in the formation of larger microspheres that could trap higher concentrations of the compound. This is because the increased amounts of active Ca^2+^-binding sites in the linear polymer resulted in a higher number of cross-links as the amount of the sodium salt of the alginic acid increased [47]. The microspheres were characterized by the SEM/EDX, XRD, and FTIR techniques. SEM images of the TiO_2_ nanoparticles showed that the particles were uniform in size, spherical, and free of clumping (see Figure 5) [62]. The surface roughness was more pronounced and cracks were evident in the CI-TiO_2_-MSs (see Figure 7), whereas a smooth surface was observed in the unloaded microspheres (see Figure 6). A cross-sectional SEM micrograph of the CI-TiO_2_-MSs also showed that they were fully loaded with the TiO_2_ nanoparticles and essential oil (see Figure 7C, D and Figure 8C, D) [63,64]. The XRD pattern of the TiO_2_ nanoparticles was examined. According to Figure 8A, a well-crystallized rutile profile was observed for the TiO_2_ nanoparticles because the peaks were sharp. Figure 8B also shows the XRD patterns of the sodium salt of alginic acid, which is crystal-like because of its strong intermolecular hydrogen bonds between the chains of alginate [64]. Three diffraction points, at 111, 220, and 311, were identified for the blank microspheres (MSs); they resulted from the surface reflections of α1-guluronate residue at 111 and the β-D-mannuronate residue at 220 [65]. Figure 8C shows the XRD pattern of the CI-TiO_2_-MSs as containing rutile peaks, as well as peaks of calcium alginate. The FTIR spectrums of the TiO_2_ nanoparticles and modified microspheres are seen in Figure 9. The FTIR spectrum was used for the chemical interpretation of the synthesized TiO_2_ nanoparticles and modified microspheres. These spectrums are shown in Figure 9. The peak values and functional group vibrational modes of the microspheres and TiO_2_ nanoparticles are given in Table 1 [42,43,44,45,46]. A bending vibration of 3416–2360 cm^−1^ is characteristic of the -OH group (which is due to water molecules), and 666 and 480–521 cm^−1^ are characteristic peaks of rutile phase TiO_2_ nanoparticles. The -OH bending mode of water Ti-OH is 1642 cm^−1^. The asymmetric stretching vibration of free carboxyl groups is 1594 cm^−1^, and the stretching of the C-O-C bond in unloaded microspheres is 1017 cm^−1^. The absorption stretching of the aromatic benzene ring is 1610 cm^−1^. The stretching vibration of an aldehyde group is 1500 cm^−1^. The vibrational mode of a phenyl ring is 1442 cm^−1^, that of a –CH_3_ group is 1376 cm^−1^, and that of the –C-O-C of aromatics is 1262 cm^−1^.

In this research, TiO_2_ nanoparticles were synthesized, which were spherical, crystalline, and in the rutile phase. The TiO_2_ nanoparticles were of uniform size. The synthesized CI-TiO_2_-MSs were fully loaded with an essential oil and the TiO_2_ nanoparticles, as was evident from the SEM/EDX, XRD, and FTIR findings. The CI-TiO_2_-MSs had strong antibacterial potency, as was evident from the antibacterial activity assay. Further, the flow cytometry confirmed cell death from the CI-TiO_2_-MSs because the cell population in the right upper quadrant had cells in the late apoptotic phase in the CI-TiO_2_-MSs. It was confirmed that the cell death caused by the CI-TiO_2_-MSs showed late apoptotic features possibly resulting in cell damage. The SEM micrograph of *S. aureus* treated with CI-TiO_2_-MSs also confirmed cell death. Cytotoxicity analysis showed that the CI-TiO_2_-MSs did not cause the hemolysis of the red blood cells incorporated in the agar plates. Biofilm analysis depicted the successful inhibition of biofilm formation by the CI-TiO_2_-MSs.

### Proposed Mechanism of Action of Modified Microspheres

**Figure 15:**
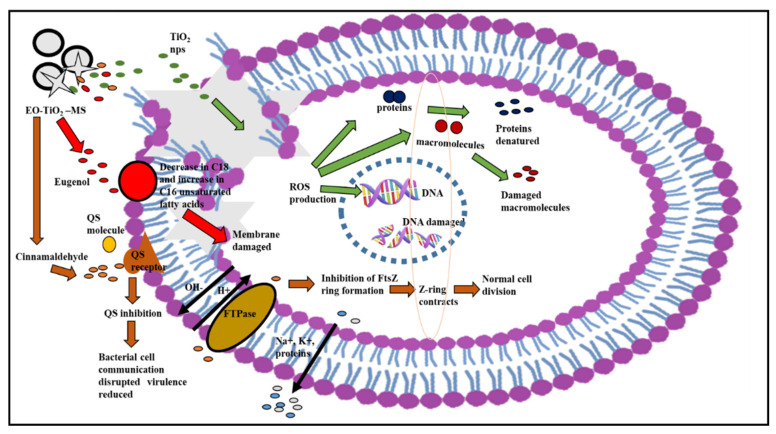
Proposed mechanism of antibacterial action of CI-TiO_2_-MS on *S. aureus* [68,69,70].

The CI-TiO_2_-MSs had a positive response against *S. aureus* (Figure 15). The CI-TiO_2_-MSs released the essential oil and TiO_2_ nanoparticles and damaged the cell membrane, entered the cell, and led to the production of ROS, which further caused DNA damage, protein denaturation, and the destruction of macromolecules [66]. Key components of cinnamon essential oil are cinnamaldehyde and eugenol. The essential oil components can affect both the outer membrane of a cell and the cell’s cytoplasm. Essential oils caused a loss of the cell’s outer membrane, altered the amount of fatty acids in the cell membrane, inhibited the cytoplasmic and cell division proteins, inhibited the ATP synthesis enzyme, and inhibited the quorum sensing. Eugenol damages the outer membrane by reducing the electron-thick material from the inner bilayer of the cell membrane. The lipophilic constituents of essential oils alter the cell morphology by disturbing the amounts of unsaturated fatty acids. The flexibility and rigidity of a cell are destroyed as the proportion of saturated fatty acids is augmented in the membrane’s bilayer. Cinnamon might directly affect the cell membrane or its fatty acid metabolism and inhibit the growth of *Lactobacillus acidophilus* by lowering the amount of unsaturated fatty acids and cyclopropane fatty acids. Similarly, thymol, eugenol, and carvacrol altered unsaturated fatty acids (stearic acid; C_18_) and saturated fatty acids (hexadecanoic acid; C_16_) [62,67]. Cinnamaldehyde hinders cell separation by decreasing the contact between the cell division site and FtsZ, a tubulin-like protein important for cell division. It also disrupts the long-chain formation of the FtsZ protein [63]. Eugenol disrupts cellular mechanisms by inhibiting cytoplasmic membrane enzymes and becomes encircled by the molecules of the lipids in *Listeria monocytogenes* and *Escherichia coli* [68]. Different enzymes were inhibited by this, including ATPases, protease, α-amylase, carboxylases, and histidine [64]. Essential oils cause the membrane bilayer to disintegrate by disrupting the stability between the intracellular and extracellular ATPs. The damaged membranes lead to the loss of ATP and cell disruption. When *S. aureus* is treated with mustard essential oil, ATP from the inside of the cell is lost [65]. Cinnamon constrains the cell wall synthesis enzymes [58]. The cinnamon essential oil established the antiquorum sensing activity on mutant and wild strains. The basic mechanism of quorum sensing includes the inhibition of signals and the biosynthesis activity of AHL-producing enzymes, the degradation of enzymatic signals, and the inhibition of signal receptor molecules [59].

## 5. Conclusions

TiO_2_ nanoparticles were prepared using an extract of the *N. sativa* seed. Microspheres were produced by the ionotropic gelation method. They were spherical, of uniform size, and microporous and had a rough surface. The synthesized microspheres were fully loaded with an essential oil and TiO_2_ nanoparticles. Both the TiO_2_ nanoparticles and the microspheres were characterized by the SEM/EDX, FTIR, and XRD techniques. The results showed that the TiO_2_ nanoparticles were in the nanometer range and spherical in shape, whereas the CI-TiO_2_-MSs were spherical and had a rough surface. Apoptosis analysis and SEM micrographs of *S. aureus* cells treated with CI-TiO_2_-MSs showed that they had strong bactericidal activity and antibiofilm activity. The CI-TIO2-MSs had no toxic impact on human erythrocytes. As a result, employing these microspheres to produce novel active alternative antibacterial delivery materials for suppressing *S. aureus* could be a potentially useful method.

## Figures and Tables

**Table 1:** Peak values and functional group vibrational modes obtained from microspheres and TiO_2_ nanoparticles.

Wave Number (cm^−1^)	Vibration Mode	References
480–521	Ti-O-Ti bending vibration	[42]
664	Ti-O-Ti bending vibration	[42]
1631	OH mode bending mode of water Ti-OH	[43]
1595	Asymmetric stretching vibrations of free carboxyl groups	[44]
325	OH stretching peak	[44]
1020	Stretching of C-O-C bond	[44]
1605	Stretching absorption of benzene ring	[45]
1510	Stretching C=O of aldehyde group	[45]
1431	Phenyl ring	[46]
1366	-CH_3_	[46]
1266	=C-O-C of aromatics	[46]

## Data Availability

Not applicable.

## Abbreviations

AHL	Acyl homoserine lactone
ATP	Adenosine triphosphate
ATPase	adenosine triphosphatase
CaCl_2_	Calcium chloride
CI	Cinnamon essential oil
CI-TiO_2_-MS	Microspheres loaded with cinnamon essential oil and TiO_2_ nanoparticles
CLSI	Clinical and Laboratory Standards Institute
DMSO	Dimethylsulfoxide
DNA	Deoxyribonucleic acid
EDX	Energy dispersive X-ray
FITC	fluorescein isothiocyante
FTIR	Fourier transform infrared
FtsZ	Filamenting temperature-sensitive mutant Z
GCMS	Gas Chromatography Mass Spectrometry
MDR	Multi-drug resistant
MS	Blank microsphere
PBS	Phosphate buffer saline
ROS	Reactive oxygen species
SEM	Scanning electron microscopy
TiO_2_-MS	Microspheres loaded with TiO_2_ nanoparticles
XRD	X-ray Diffraction
